# Histopathological examination of appendicectomy specimens at a district hospital of Saudi Arabia

**DOI:** 10.12669/pjms.314.7453

**Published:** 2015

**Authors:** Mohammad Ayub Jat, Farhan Khashim Al-Swailmi, Yasir Mehmood, Majid Alrowaili, Shehab Alanazi

**Affiliations:** 1 Dr. Mohammad Ayub Jat, FCPS. Assistant Professor of Surgery, College of Medicine, Northern Border University, Arar, Saudi Arabia; 2 Dr. Farhan Khashim Al-Swailmi, MD. Assistant Professor of Ophthalmology. Head of Department of Surgery, College of Medicine, Northern Border University, Arar, Saudi Arabia; 3 Dr. Yasir Mehmood, FCPS. Assistant Professor of Surgery, College of Medicine, Northern Border University, Arar, Saudi Arabia; 4 Dr. Majid Alrowaili, MD. Assistant Professor of Orthopaedic, College of Medicine, Northern Border University, Arar, Saudi Arabia; 5 Dr. Shehab Alanazi, Dean of Northern Border University, College of Medicine, Northern Border University, Arar, Saudi Arabia

**Keywords:** Appendicitis, Appendicectomy, Histopathology of appendix specimen

## Abstract

**Background and Objective:**

Appendicectomy for acute appendicitis is one of the most commonly performed surgical procedures. Acute appendicitis has remained a clinical entity and an ongoing diagnostic challenge. A retrospective study was performed to determine histological diagnosis, demographic data, the rate of perforated appendicitis and negative appendicectomies.

**Methods:**

Histopathological records of 480 resected appendices submitted to histopathology department at Arar Central Hospital in the Northern Border Province of Saudi Arabia over the period of 3 years from July 2011 to June 2014 were reviewed retrospectively, to determine acute appendicitis, complication (gangrene, perforation) rate, negative appendicectomy rate, histopathological diagnosis and unusual finding on histology.

**Results:**

Out of 480 specimens of appendix, appendicitis accounted for 466 (97.0%) with peak occurrence in the age group of 11 to 50 years in male and 11 to 40 years in female. Histopathological diagnosis include acute appendicitis 250 (52.0%), suppurative appendicitis 135 (28.0%) acute gangrenous appendicitis 60 (12.5%), perforated appendicitis 9 (2.0%), chronic appendicitis 12 (2.5%). Negative appendicectomy rate was 14 (3%) and two time more common in female with peak occurrence in the age group of 20-30 yrs. There was no unusual histological finding like carcinoid tumour of appendix.

**Conclusion:**

The present study showed a high number of appendicitis in adolescents and young adults. Negative appendectomy was more common in females. The study support routine histological examination of all the appendicectomy specimens to avoid missing of any clinically important and treatable condition.

## INTRODUCTION

Acute appendicitis is the most common general surgical emergency. The lifetime risk for appendicitis is 7%, commonly occurring in adolescent and young adults.^1^ The rate of acute appendicitis varies among countries. In USA and Europe there is a declining rates of acute appendicitis.^2^ In developing countries due to changes in life style there is increasing incidence in most urban centers.^3^ Despite of advances in technology and imaging modalities, there is dilemma in the diagnosis of acute appendicitis, however histopathological examination still remains the gold standard for the confirmation of appendicitis. There is evidence that intra-operative normal appendices may have abnormal incidental finding at cytology level and the practice of sending appendicectomy specimens for routine histopathological examination differs between centers as well as the literatures.^4^

This study aimed to determine the various histological diagnoses of all surgically removed appendices and to find out the age and sex related occurrence of appendicitis, the perforation rate and the rate of negative appendectomies.

## METHODS

The present retrospective study was conducted in the Surgical Department Arar Central Hospital in Northern Border Province of Saudi Arabia during the three years period from July 2011 to June 2014. All emergency appendectomies and interval appendectomies performed on clinically suspected appendicitis were included. All appendicectomies were performed by open technique under general anaesthesia. All the surgically resected appendices submitted to Department of Pathology were included in this study. Exclusion criteria included patients who underwent concomitant resection of other intra abdominal organs such as Right hemicolectomy.

Relevant clinical data retrieved included patients’ age, gender, pre-operative clinical presentation and operative findings. Various Histopathological diagnoses obtained from the appendicectomy specimens were retrieved from histopathological record registers and an assessment was made of whether histopathological diagnosis caused any alteration in the patient’s subsequent management or not. In acute appendicitis there are neutrophils in mucosa, wall of appendix and congested blood vessel with fibrinous exudates in the serosa while suppurative appendicitis associated with obstruction (fecalith, ball of worms) and abscess formation within the wall and foci of suppurative necrosis in the mucosa.^5^

Negative appendectomy was defined as one which is performed for a clinical diagnosis of acute appendicitis but in which the appendix is found to be normal on histopathological examination.

## RESULTS

Total of 480 specimens of appendix were received in the histopathology department at Arar Central Hospital during the period of 3 years from July 2011 to June 2014 Out of total 480 appendectomies there were 460 emergency appendectomy and 20 interval appendectomy performed for clinically suspicious appendicitis. In this study 466 (97.0%) cases were found to have histologically proven appendicitis and 14 (3%) cases were normal appendix. There were 280 (58.3%) males and 200 (41.7%) females, with the male: female ratio of 1.4:1. The mean age for male was 28 years with a range of 11 to 50 yrs. The mean age for female was 20.5 years with a range of 11 to 40 yrs. However male 247 cases (88%) with age group 11 to 50 years and in female 168 cases (84%) with age group 11 to 40 years. The distribution of acute appendicitis according to age group is shown in Table-I.

**Table-I:** The distribution of acute appendicitis according to age group.

Age Group	Male 280 (58.3%)		Female 200 (41.7%)	
< 10 YR	18	6.5%	10	5%
11 – 20	100	35.7%	80	40%
21 – 30	75	26.8%	58	29%
31 – 40	50	17.8%	33	16.5%
41 – 50	22	7.8%	15	7.5%
⊳ 51	15	5.4%	4	2%

The Perforated appendicitis rate was 1.8% (9 out of 480 cases) and was seen more in female at the age interval of 12 – 30 years. The rate of negative appendectomy was 3% (14 out of 480 cases) significantly more in female patients with the female: male ratio of 2:1 in the age group of 21 to 30 years. The histopathological findings showed that the occurrence of acute appendicitis was 52% followed by acute suppurative 28% and gangrenous appendicitis 12.5%. The distribution of various histopathological findings of acute appendicitis by gender is shown in Table-II.

**Table-II:** The distribution of various histopathological findings of acute appendicitis by gender.

Histological Findings	Female (n=200)		Male (n= 280)		Total (n=480)	
Acute appendicitis	47.5%	(95)	55.4%	(155)	52.0%	(250)
Suppurative appendicitis	32.5%	(65)	25.0%	(70)	28.0%	(135)
Gangrenous appendicitis	11.0%	(22)	13.5%	(38)	12.5%	(60)
Perforated appendicitis	2.5%	(5)	1.5%	(4)	2.0%	(9)
Chronic appendicitis	2.5%	(5)	2.5%	(7)	2.5%	(12)
Normal appendix	4%	(8)	2.1%	(6)	3%	(14)

## DISCUSSION

Acute appendicitis has been the most common surgical emergency and appendectomy is the most frequently performed abdominal operation. In western world acute appendicitis account for about 40% of all surgical emergencies.^6^ Study at South Korea done by Lee et al., showed an overall incidence of appendicitis and appendectomy of 22.71 and 13.56 per 10,000 population per year, respectively which was found to be higher than that of western countries, i.e. 7.5 to 12.0 per 10,000 population per year.^7^ Study from Nepalgunj by Khan et al.,^8^ reported acute appendicitis to be the most common cause of emergency laparotomy showing incidence of 26%.

About 80% of appendicitis occurre below 40 years of age in concordance with various studies,.^9^, In our study acute appendicitis was higher in the age group of second and third decade and about 85% of appendicitis occurring below 40 year of age. Some authors have reported a sex difference for appendicitis with male being more common than female. Some studies showed male to female ratio ranging from 1.1 to 2.9:1.^10^ Study by SalehAl–Mulhim showed 540(61.2%) patients were males and 38.8% were female, with a ratio 1.6:1.^11^ Study by Shrestha R et al. had slightly female preponderance with the female male ratio of 1.1:1.^12^ The present study showed 280 (58.3%) patients were male and 200 (41.7%) were female with a male: female ratio 1.4:1. There male preponderance could be because of majority of male adults coming from different part of the world in search of work to Saudi Arabia and might have disturbed the overall population structure.

The present study showed that frequency of histopathological diagnosis was acute appendicitis (52.%) followed by acute suppurative (28%), acute gangrenous appendicitis (12.5%), acute perforated appendicitis (2%), resolving or chronic appendicitis (2.5%) similar results were also showed in other studies done by Shrestha R and Zulfikar et al.^12^,^13^ The perforation rate of appendix in this study was low (2%) similar to that shown by other studies.^15^ The low perforation rate might be due to early visit in the surgical clinic and the prompt decision to operate for suspected appendicitis by the surgeons. Low perforation rate indicate a better prospective regarding morbidity and mortality. The study showed Negative appendicectomy rate was 3% (14) cases, with female having higher rate frequently occurring within the younger age group of 11 to 30 yrs. The negative appendectomy rate falls within the acceptable range of 10 to 20%.^6^ Various studies,^14^,^15^ has shown a wide range of rate that falls between 6.1 to 34.2%, with higher value in female. Therefore, especially in females other causes of abdominal pain should be searched out if the appendix appears normal during operation.

In this study we found other pathologies such as twisted ovarian cyst, follicular cyst and Meckel’s diverticulitis as the causes of acute abdominal pain in case of negative appendectomy, as also showed in other studies.^16^ The role of diagnostic laparoscopy followed by appendectomy if necessary in fertile female patients was found to reduce the rate of negative appendicectomy manifolds.^17^ A significant number of histologically normal appendix specimens (22.5%) showed increased cytokines expression, indicating an inflammatory reaction, therefore normal looking appendices have a 22% chance of being inflamed on further sophisticated investigations. Wang et al.^18^ demonstrated that TNF –α and interleukin - 2 expression are sensitive markers of inflammation in appendicitis.

In the present study, there was not a single case of incidental carcinoid tumour of appendix. The literature review^19^ showed the incidence of carcinoid tumour ranging from 0.1% to 1.05% mostly found incidentally during microscopic examination.

## CONCLUSION

The present study showed that histopathological examination of the appendix yield important clinical information in addition to the operative findings and should be undertaken in all cases of acute appendicitis.
